# Antiangiogenic therapy with recombinant human endostatin may improve blood perfusion of cervical node with necrosis in nasopharyngeal carcinoma patients: a preliminary study by using contrast-enhanced ultrasound

**DOI:** 10.3389/fonc.2025.1521762

**Published:** 2025-01-31

**Authors:** Zhendong Yang, Huimin Xiao, Xigui Li, Zhuxin Wei, Min Kang, Rensheng Wang, Jianyuan Huang

**Affiliations:** ^1^ Department of Radiation Oncology, The First Affiliated Hospital of Guangxi Medical University, Nanning, China; ^2^ Department of Oncology, Rui-Kang Hospital Affiliated to Guangxi University of Chinese Medicine, Nanning, China; ^3^ Department of Ultrasonography, The First Affiliated Hospital of Guangxi Medical University, Nanning, China

**Keywords:** antiangiogenic therapy, blood perfusion, contrast-enhanced ultrasound, nasopharyngeal carcinoma, cervical node with necrosis

## Abstract

**Background:**

The cervical node with necrosis (CNN) is an important poor prognostic factor for nasopharyngeal carcinoma (NPC) patients. The tumor microenvironment of the CNN has severely insufficient blood perfusion, thus leading to hypoxia and reducing the effect of radiotherapy (RT) and chemotherapy. By using contrast-enhanced ultrasound (CEUS) as a monitoring method, we conducted this study to assess whether antiangiogenic therapy (AT) with recombinant human endostatin (RHES) may improve blood perfusion of the CNN.

**Materials and methods:**

Fifteen NPC patients with CNN were enrolled and underwent CEUS the day before and day 5 after AT with RHES initiation, respectively. By analyzing the variations of CEUS parameters of CNN, such as peak intensity (PI), time to peak (TTP), and mean transit time (MTT) at different time points, we evaluate the impact of AT with RHES on blood perfusion of CNN.

**Results:**

The PI of day 5 after AT was significantly enhanced compared to the PI of the day before AT [−44.94 ± 4.72 (dB) vs. −50.33 ± 6.85 (dB), *p* < 0.001]. The TTP of day 5 after AT became dramatically shorter than the TTP of the day before AT [19.48 ± 3.63 (s) vs. 24.19 ± 6.93 (s), *p* = 0.031]. The MTT of day 5 after AT became obviously shorter than the MTT of the day before AT [28.08 ± 3.03 (s) vs. 33.76 ± 6.20 (s), *p* = 0.001].

**Conclusion:**

These results revealed that the blood volume and the blood flow velocity in the microvessels of the CNN increased after AT, indicating that AT with RHES may improve blood perfusion in the CNN of NPC, thus providing valuable insights for the clinical application of AT combined with RT and/or chemotherapy in NPC patients with CNN. Moreover, CEUS as a noninvasive and real-time monitoring method may be suitable for clinically evaluating tumor blood perfusion changes.

## Introduction

Nasopharyngeal carcinoma (NPC) is one of the most common malignant tumors in East Asia, Southeast Asia, and North Africa (1). For NPC, intensity modulated radiotherapy (IMRT) combined with chemotherapy is the primary and most effective treatment, resulting in significant improvements of the therapeutic effect compared with the era of two-dimensional radiotherapy (RT) (2). However, local recurrence and/or distant metastasis after IMRT occurs in a subset of NPC patients. The risk factors include advanced clinical staging and not receiving chemotherapy (3). In addition, the metastatic cervical node with necrosis (CNN) is also a poor prognostic factor that should be nonnegligible. Recently, Luo (4) essentially generalized the complex relationship between NPC cells and the tumor microenvironment (TME), advocating a novel perspective that NPC should be considered as a complex ecological disease—a multidimensional spatiotemporal pathological ecosystem rather than a genetic disease. Through the “Mulberry-fish-ponds” ecological model, Luo explained the dynamic interaction and co-evolution between tumor cells and TME. The pathological ecosystem of NPC, especially the TME of the CNN, has severely insufficient blood perfusion, thus leading to hypoxia, making the tumor more aggressive and antagonizing the effect of RT and chemotherapy, and leading to negative impacts on the prognosis of NPC (5–7). Several clinical studies have shown that the addition of antiangiogenic therapy (AT) to RT and chemotherapy was an effective regimen for advanced NPC (8–10), and the “vascular normalization” plays a crucial role in optimizing the synergistic effect of such combination therapy (11, 12).

In a previous study using contrast-enhanced ultrasound (CEUS), Yang et al. (13) reported that AT with recombinant human endostatin (RHES) had a vascular normalization effect on NPC patients and improved the blood perfusion of the primary tumors in nasopharynx within 5 days. Nevertheless, the CNN had worse vascular abnormality, whether AT with RHES can improve blood perfusion of the CNN and alleviate hypoxia, thereby enhancing the therapeutic effect of NPC patients with CNN, thus necessitating further research. Consequently, this study aims to utilize CEUS to observe and analyze the variations in CEUS parameters of the CNN before and after AT with RHES and then explore the impact of AT on blood perfusion of the CNN and provide evidence supporting AT treatment in NPC patients with CNN.

## Material and methods

### Patients

A total of 15 NPC patients diagnosed by histopathology in the First Affiliated Hospital of Guangxi Medical University from August 2019 to August 2020 were enrolled in this study. The inclusion criteria were as follows (1): diagnosed with histopathology-confirmed NPC (2); without any previous anti-tumor treatment; (3) stage III–IVA NPC according to the 8th edition of the UICC/AJCC staging system; (4) metastasis of CNN diagnosed by magnetic resonance imaging (MRI); (5) Karnofsky scores ≥70; and (6) signed informed consent.

The exclusion criteria were as follows: (1) age <18 years or >70 years; (2) allergic to SonoVue (Bracco, Milan, Italy); (3) not suitable for MRI; (4) cervical vertebra injury; (5) infection or rupture in neck skin; (6) cardiovascular right to left shunt; (7) severe cardiac arrhythmias; (8) myocardial infarction; (9) severe pulmonary hypertension; (10) hemorrhagic tendency; (11) pregnancy or lactation; and (12) mental disorder.

This study was carried out in accordance with the Declaration of Helsinki of 1975, revised in 2008. Ethical approval (no. 2018[KY-E-042]) was obtained from the medical ethics committees of the First Affiliated Hospital of Guangxi Medical University. All patients signed informed consent and all patient details were de-identified.

### Selecting the target CNN

**Figure 1:**
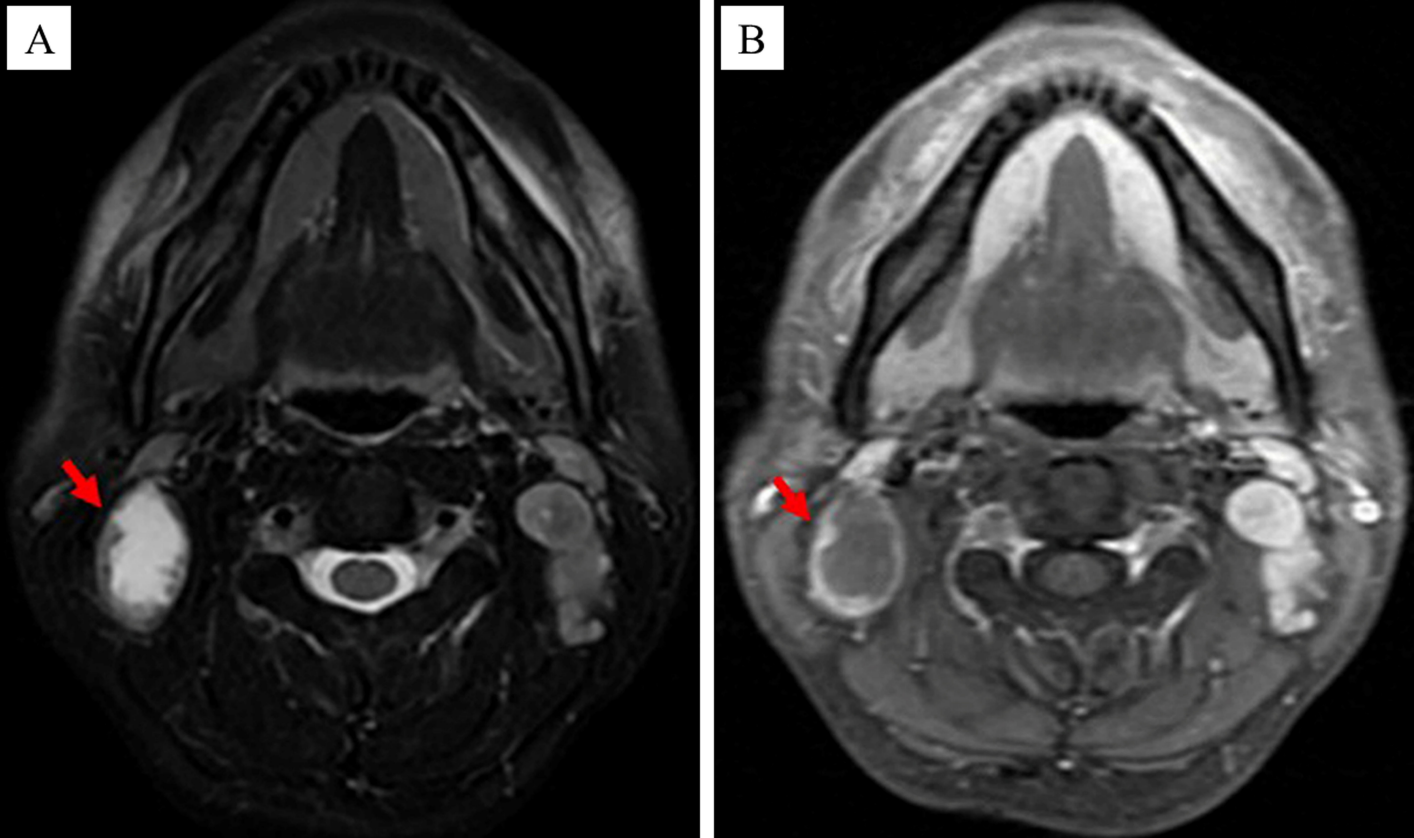
MRI images of the CNN (as indicated by the red arrow). **(A)** The central region of the CNN showed high signal on the T2-weighted sequence. **(B)** The central region of the CNN showed low signal on the T1 enhancement sequence.

**Figure 2:**
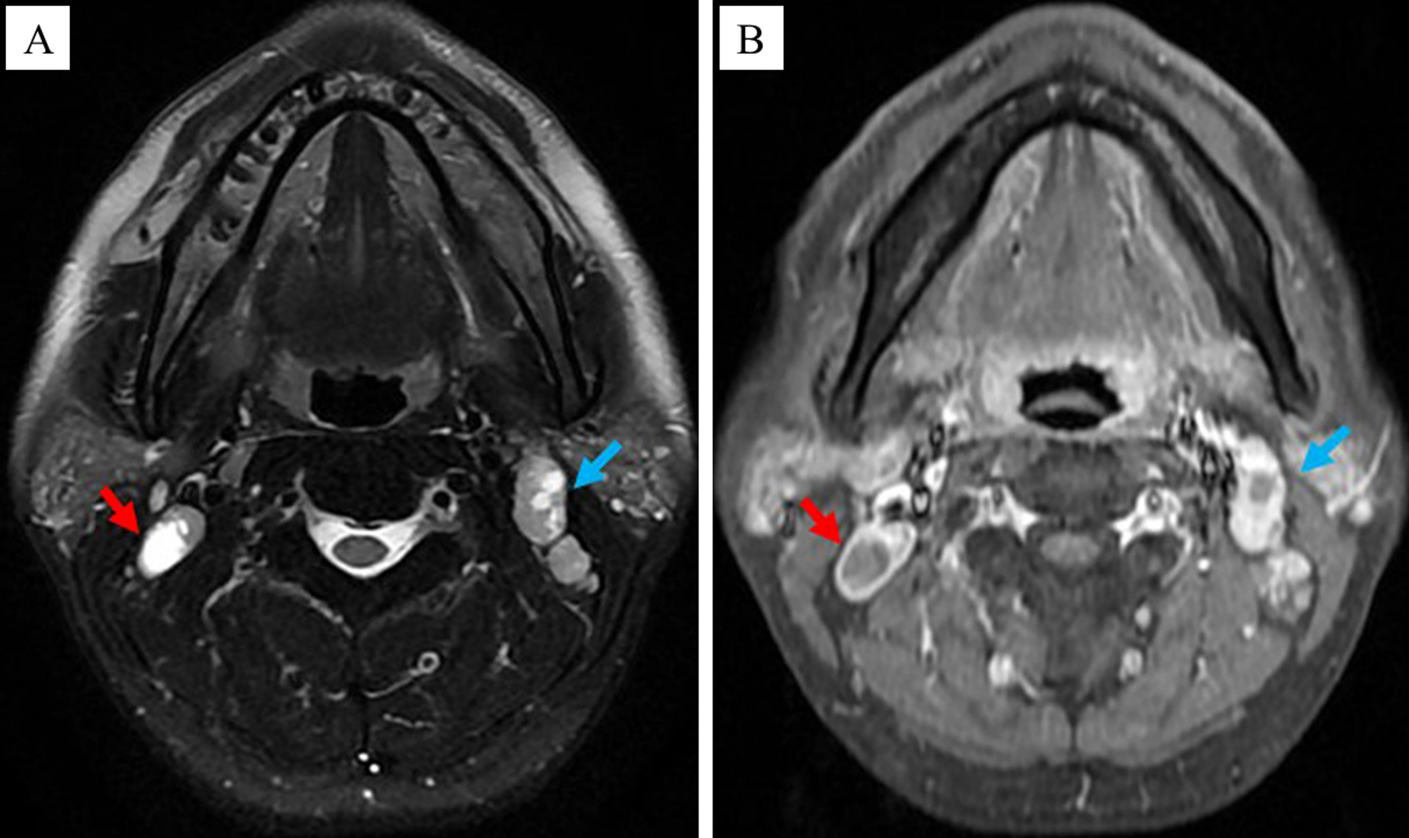
Principle of selecting the tCNN. If multiple CNNs were present in the same patient, the one with the largest proportion of necrotic area, as indicated by the red arrow rather than the green arrow, should be selected. **(A)** MRI images of the tCNN on the axial T2 sequence. **(B)** MRI images of the tCNN on the axial T1 enhancement sequence.

Each patient enrolled in this study underwent dynamic contrast-enhanced MRI of the nasopharynx and neck prior to the treatment, and some specific lymph nodes were selected as the target CNN (tCNN). The MRI features of CNN were as follows (14): The central region of the lymph node showed high signal on the T2-weighted sequence and low signal on the T1 enhancement sequence, with or without marginal reinforcement (**Figures 1A, B**). If multiple CNNs were present in the same patient, the one with the largest proportion of necrotic area (i.e., on the axial T2 sequence, the long diameter of the necrotic area divided by the long diameter of the TLNs) should be selected (**Figures 2A, B**).

### AT with RHES

All patients received AT with RHES (solubilized in 250 mL of 0.9% normal saline) intravenously with a dosage of 7.5 mg/m^2^ per day for 5 days prior to RT or chemotherapy.

### CEUS and parametric analysis

All patients underwent CEUS the day before and day 5 after AT with RHES initiation, respectively. To ensure the accuracy and consistency of CEUS, the following principles were adhered to (1): all examinations were performed by a single sonographer with over 5 years of experience in CEUS; (2) all examinations were conducted using a single Aplio 500 Ultrasound System (Toshiba, Tokyo, Japan); and (3) SonoVue (Bracco, Milan, Italy) was used as the ultrasound contrast agent.

**Figure 3:**
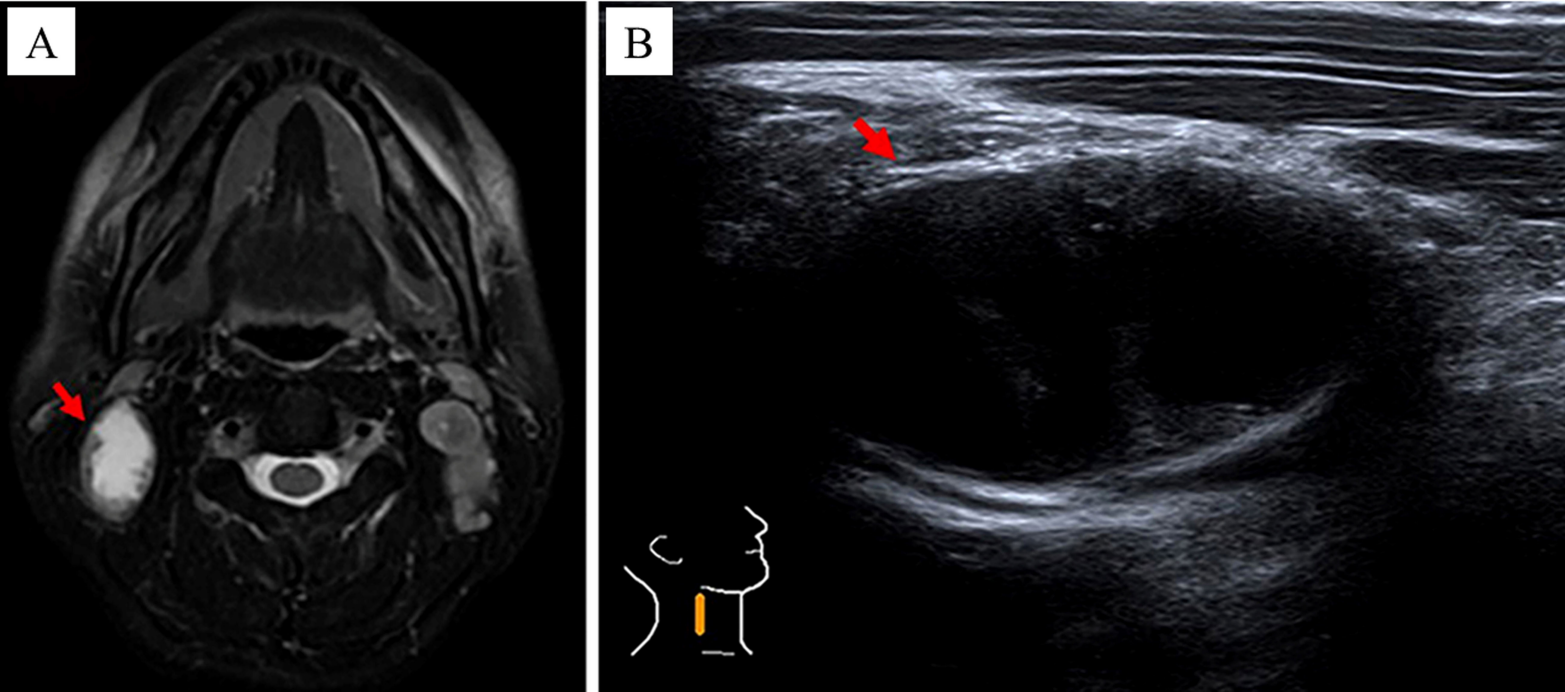
The method of locating the tCNN. In a 37-year-old male NPC patient, by referring to **(A)** MRI images, the **(B)** two-dimensional ultrasound was performed to locate the tCNN, as indicated by the red arrow.

The procedural steps for CEUS were as follows: (1) body position: the patient was positioned supine on the examination bed with hands resting naturally at their sides, and the head turned towards the opposite side of the TLN, ensuring that the opposite auricle was in close proximity to the bed surface. (2) Localization of tCNN: utilizing MRI images as a reference, two-dimensional ultrasound was performed to accurately locate the tCNN (**Figures 3A, B**). (3) Recording of parameters: detailed documentation of body position, probe angle, initial location, and depth of tCNN during first CEUS procedure, along with other relevant technical parameters of the ultrasound examination system for future reference during subsequent CEUS procedures. (4) Preparation and administration of contrast agent: SonoVue solution was prepared by dissolving one bottle in 5 mL of normal saline, which was then thoroughly mixed before injecting 2.4 mL into an indwelling needle inserted into the left median cubital vein at a rate of 1 mL/s. Following injection, an additional 5 mL of normal saline flush was administered through the same tube. (5) Image recording and storage: Simultaneously with contrast agent administration, continuous video recording capturing CEUS images lasting at least 60 s took place; these recordings were saved in audio–video interactive (AVI) format. (6) Second CEUS procedure: on day 5 after initiation of RHES treatment, a second round of CEUS imaging was conducted following identical protocols used during initial procedure regarding patient positioning, probe angle, and other recorded parameters.

**Figure 4:**
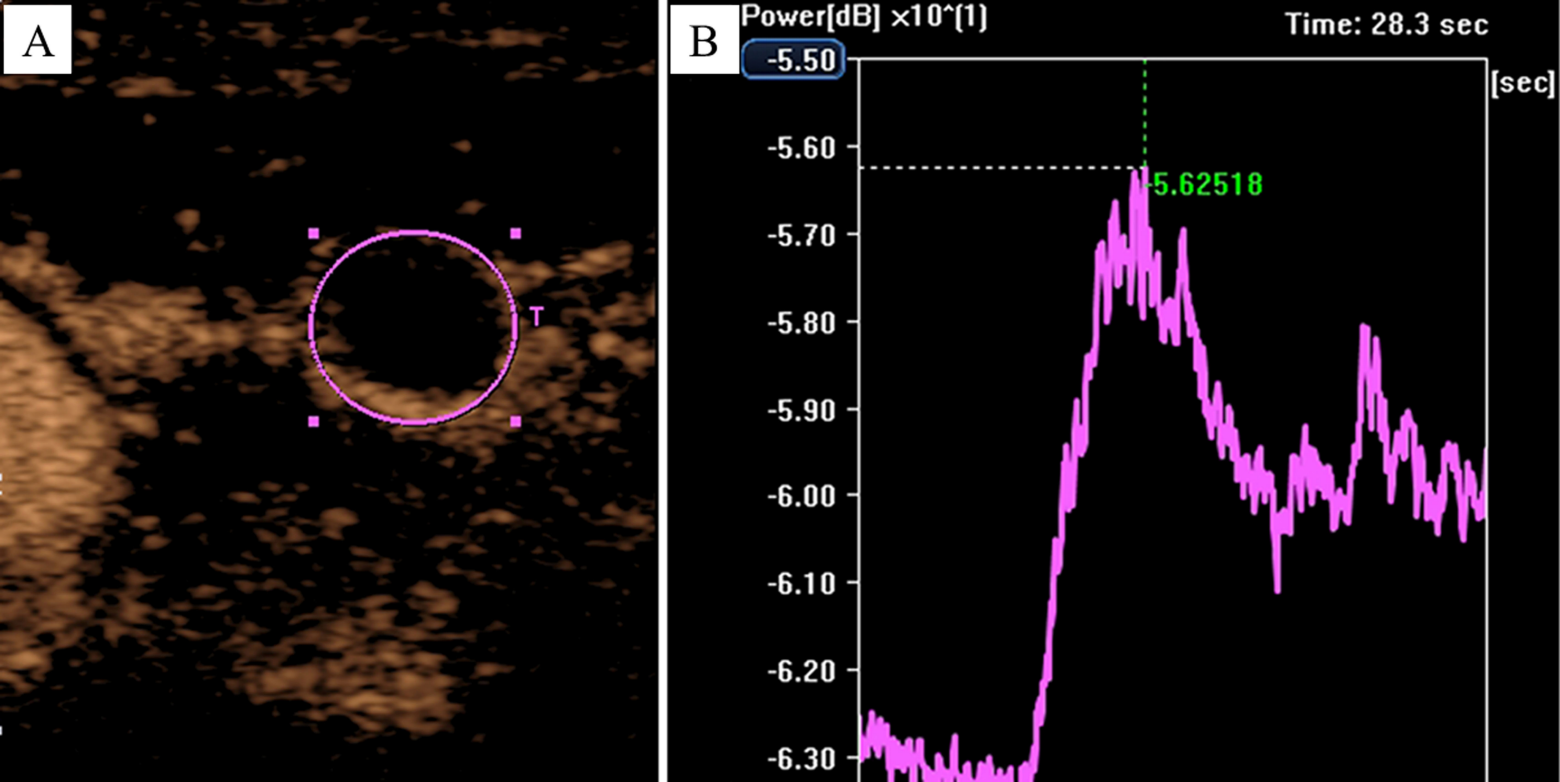
Analyzing the AVI of CEUS by using the CHI-Q software. In a 50-year-old male NPC patient, **(A)** the tCNN was set as the ROI, **(B)** then the ROI was analyzed to obtain the time/intensity curve.

Finally, the AVI of each CEUS was quantitatively analyzed using CHI-Q software. The procedure involved setting each tCNN as a region of interest (ROI) individually (**Figure 4A**). Subsequently, the ROI was analyzed to obtain the time/intensity curve (TIC) (**Figure 4B**), and various parameters such as peak intensity (PI), time to peak (TTP) and mean transit time (MTT) of each tCNN, respectively. According to relevant guidelines (15), two senior ultrasound physicians with over 5 years of CEUS experience, along with an engineer, were responsible for ensuring quality control (QC) and quality assurance (QA) during CEUS imaging and subsequent quantitative analysis.

### Statistical analysis

All statistical analyses were performed by using SPSS 23.0 (IBM Corporation, Armonk, NY, USA). The measurement data were expressed as mean ± standard deviation (SD) and analyzed by using the paired-samples *t*-test. Statistical significance was set at *p* < 0.05.

## Results

### Baseline characteristics of patients

Among the 15 patients enrolled, there were 10 male (66.7%) and 5 female patients (33.3%). The median age of these patients was 46 years (range: 34–67 years). The median height was 170 cm (range: 149–179 cm). The median weight was 70 kg (range: 45–79 kg). WHO type IIa and IIb were identified in 1 (6.7%) and 14 (93.3%) patients, respectively. According to the 8th edition of the AJCC staging system, the stage distributions were as follows: stage III, 10 (66.7%); stage IVA, 5 (33.3%) (**Table 1**).

**Table 1:** Baseline characteristics of patients.

Characteristic	Number of patients (%)
Sex	
Male	10 (66.7)
Female	5 (33.3)
Age (years)	
Median	46
Range	34–67
Height (cm)	
Median	170
Range	149–179
Weight (kg)	
Median	70
Range	45–79
Histopathology	
WHO IIa	1 (6.7)
WHO IIb	14 (93.3)
T stage	
T3	12 (80)
T4	3 (20)
N stage	
N1	4 (26.7)
N2	7 (46.7)
N3	4 (26.7)
Clinical stage	
III	10 (66.7)
IVA	5 (33.3)

### Characteristics of tCNN

Of the 15 tCNN, 3 (20%) were in left neck level II, 9 (60%) were in right neck level II, 1 (6.7%) was in left neck level III, and 2 (13.3%) were in right neck level III. The median long diameter of the tCNN was 26.5 mm (range: 13.9–40.6 mm). The median long diameter of the necrotic areas was 13.4 mm (range: 8.2–29.1 mm). The median necrosis proportion of the TLN was 54.4% (range: 30.2%–93.3%) (**Table 2**).

**Table 2:** Characteristics of tCNN.

Characteristic	Number (%)
Neck node levels	
Left II	3 (20)
Right II	9 (60)
Left III	1 (6.7)
Right III	2 (13.3)
Long diameter of the tCNNs (mm)	
Median	26.5
Range	13.9–40.6
Long diameter of the necrotic area (mm)	
Median	13.4
Range	8.2–29.1
Necrosis proportion of the tCNNs (%)	
Median	54.4
Range	30.2–93.3

tCNN, target cervical node with necrosis.

### Variations of CEUS parameters

**Figure 5:**
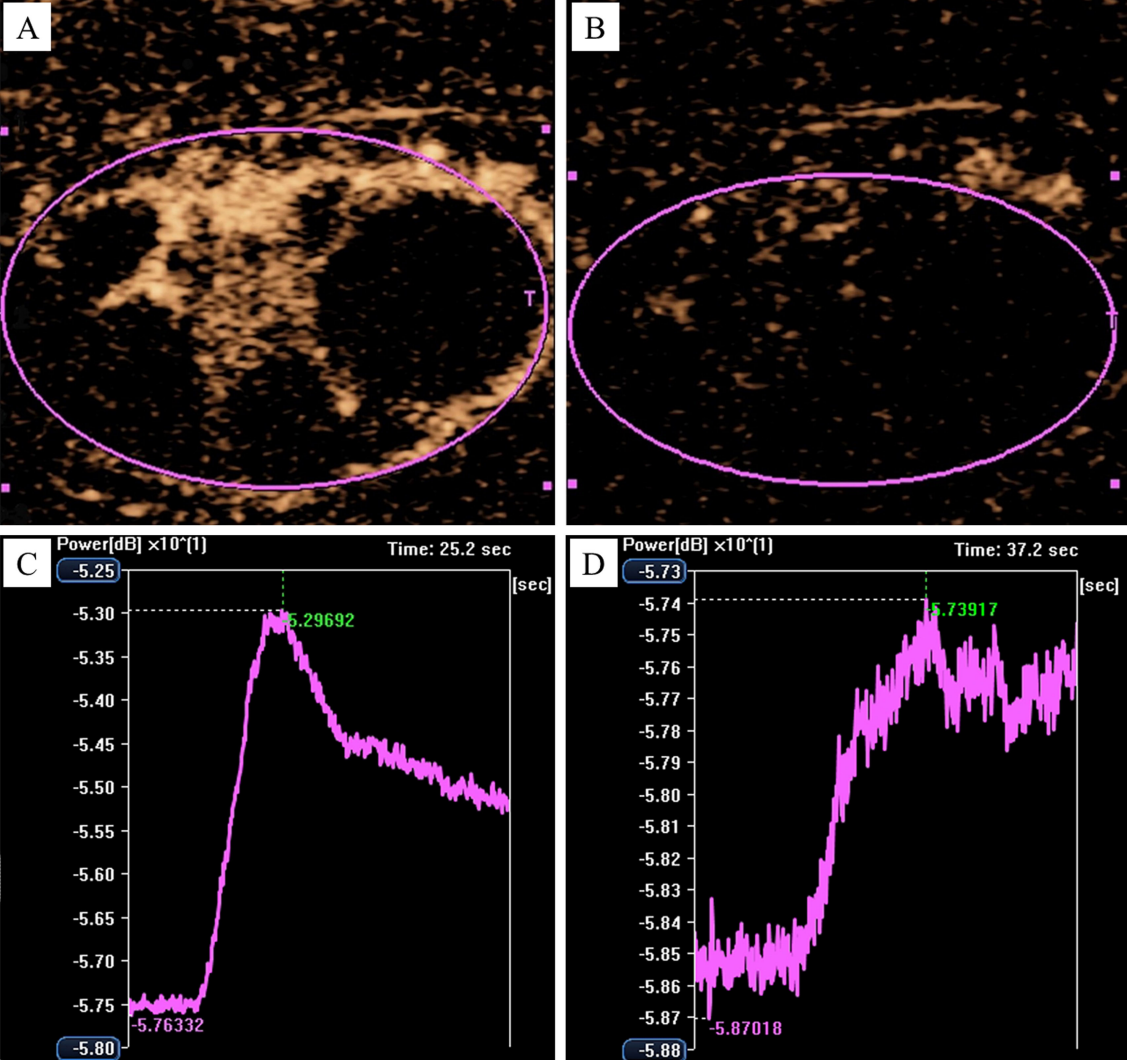
Variations of CEUS parameters before and after AT with RHES. In a 37-year-old male NPC patient, **(A)** the blood flow signal of the tCNN, especially in the central region, was dramatically increased on day 5 in comparison with **(B)** the day before RHES treatment. Furthermore, **(C)** PI on day 5 was significantly increased compared to **(D)** the day before RHES treatment.

**Figure 6:**
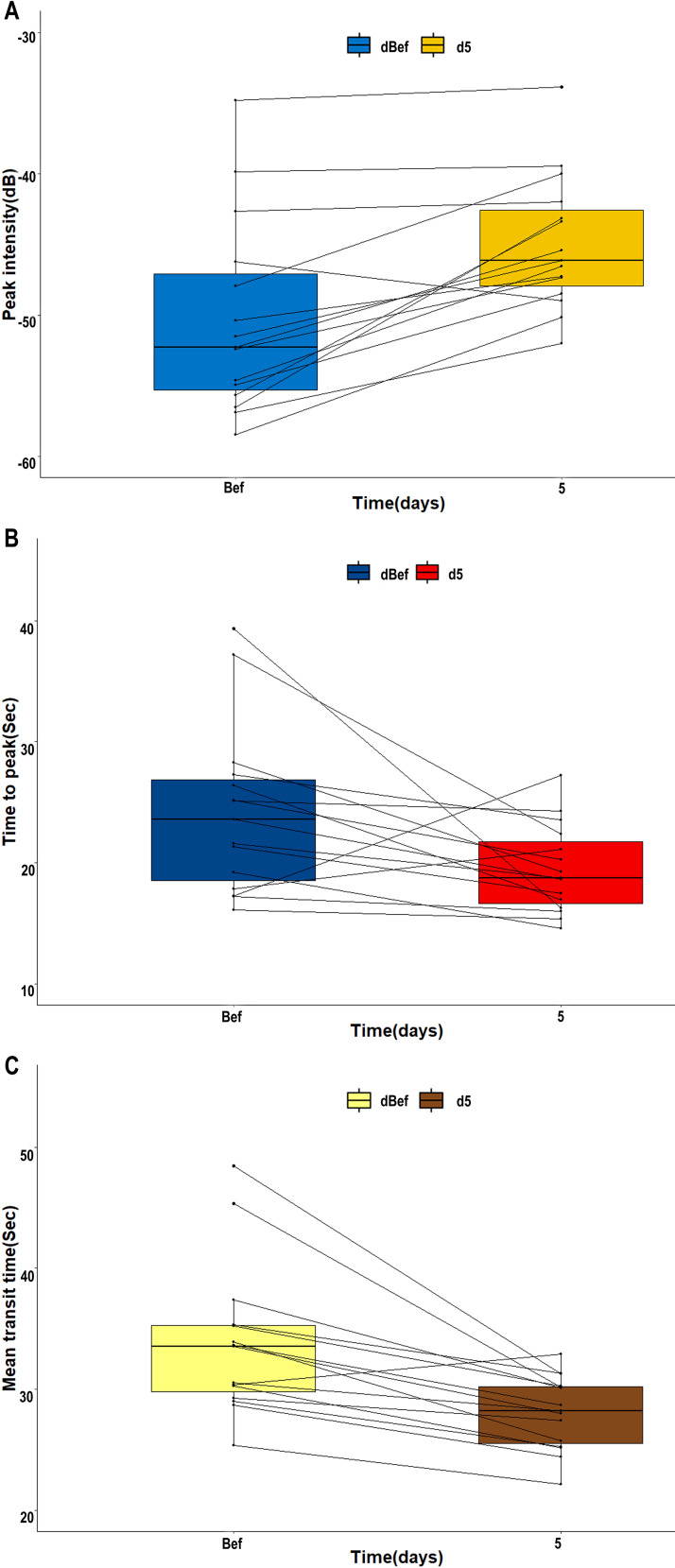
Line charts of variations of CEUS parameters of the tCNN before and after AT with RHES. In most patients, **(A)** PI_d5_ of the tCCN was enhanced compared to PI_dBef_. Meanwhile, **(B, C)** TTP_d5_ and MTT_d5_ of the tCNN were shorter than TTP_dBef_ and MTT_dBef_, respectively.

**Figure 7:**
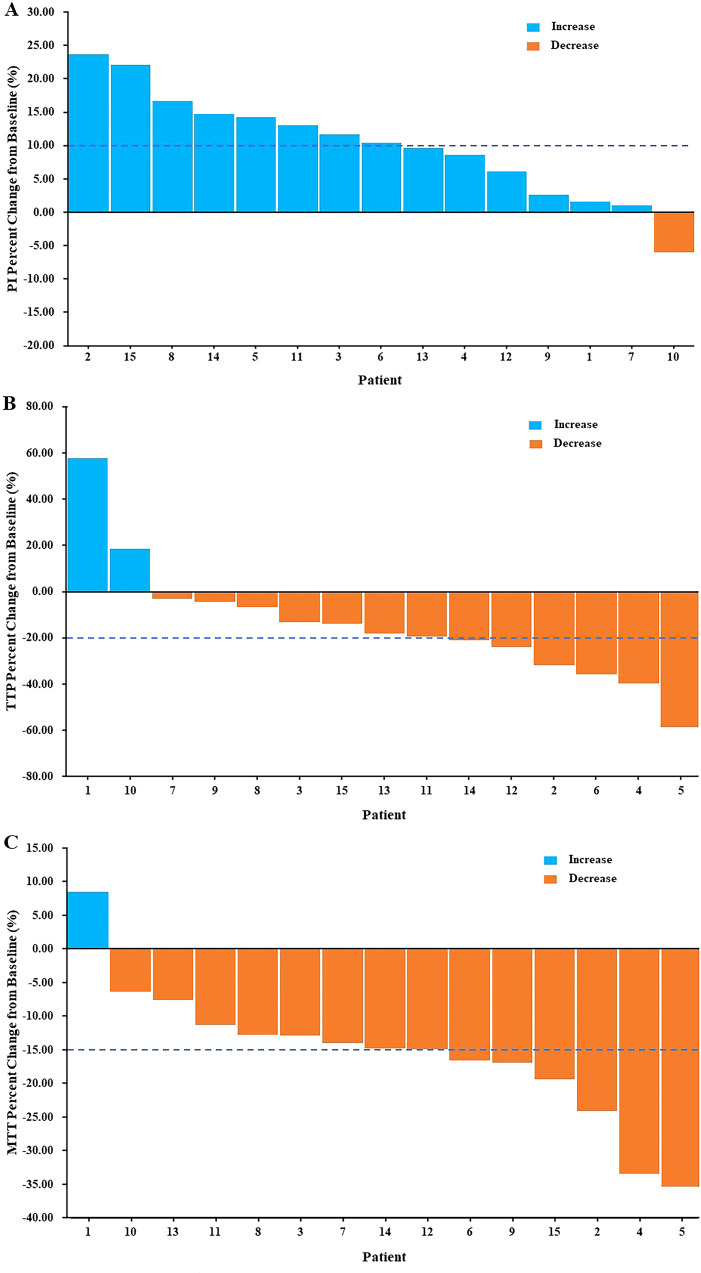
Waterfall plots of percent change in CEUS parameters of the tCNN. In comparison with the day before AT with RHES, **(A)** more than half of the patients had PI increases of more than 10%. Meanwhile, **(B)** more than a third of patients had a reduction in TTP of more than 20%. Furthermore, **(C)** nearly half of the patients had a reduction in MTT of more than 15%.

The blood flow signal of the tCNN, particularly in the central region, exhibited a significant increase on day 5 (d5) following initiation of RHES treatment when compared to the day before RHES treatment (**Figures 5A–D**). The PI of the tCNN increased in most patients after RHES treatment initiation, with only one patient demonstrating a decrease (**Figure 6A**). A total of eight patients experienced a PI increase exceeding 10% in comparison to the initiation of RHES treatment (**Figure 7A**). The mean ± SD of PI of the tCNN on d5 and the day before (dBef) RHES treatment initiation were −44.94 ± 4.72 (dB) and −50.33 ± 6.85 (dB), respectively. Obviously, PI_d5_ was significantly higher in comparison with PI_dBef_ (*p* < 0.001; **Table 3**). After RHES treatment initiation, the TTP of the tCNN decreased in most patients, but increased in only two patients (**Figure 6B**). The TTP in six patients decreased by more than 20% (**Figure 7B**). The mean ± SD of TTP_d5_ became dramatically shorter, compared with TTP_dBef_ [19.48 ± 3.63 (s) vs. 24.19 ± 6.93 (s), *p* = 0.031; **Table 3**]. Moreover, all but one patient had a decrease in MTT (**Figure 6C**). MTT in six patients decreased by more than 15% (**Figure 7C**). The mean ± SD of MTT dramatically decreased after RHES treatment initiation; MTT_d5_ and MTT_dBef_ were 28.08 ± 3.03 (s) and 33.76 ± 6.20 (s), respectively (*p* = 0.001; **Table 3**). Overall, these results revealed that the blood volume and the blood flow velocity in the microvessels of the CNN increased after AT with RHES, indicating that AT may improve blood perfusion of CNN in NPC patients.

**Table 3:** Variations of the CEUS parameters.

Parameters		Time		*t*	*p*
		dBef	d5		
PI (dB)	Mean ± SD	−50.33 ± 6.85	−44.94 ± 4.72	−4.707	<0.001
TTP (s)	Mean ± SD	24.19 ± 6.93	19.48 ± 3.63	2.404	0.031
MTT (s)	Mean ± SD	33.76 ± 6.20	28.08 ± 3.03	4.467	0.001

CEUS, contrast-enhanced ultrasound; RHES, recombinant human endostatin; dBef, the day before; RHES, treatment initiation; d5, day 5 after RHES treatment initiation; PI, peak intensity; TTP, time to peak; MTT, mean transit time; s, second.

### Adverse effect

No adverse effect related to RHES, such as allergy, headache, arrhythmia, nausea, and fever, was observed in all 15 patients.

## Discussion

The CNN is an important risk factor for treatment failure of NPC (5–7). Severe lack of blood perfusion and oxygen in the TME of the CNN reduces the sensitivity of radiation, decreases drug transportation, increases the aggressiveness of the tumor, and weakens the therapeutic effect of RT as well as chemotherapy, ultimately leading to treatment failure. Notably, several clinical studies have demonstrated that combining AT to RT and chemotherapy was effective for NPC (8, 10, 16). According to the theories proposed by Jain (11, 17) and Winkler et al. (12), AT aims not only to inhibit the formation of tumor new blood vessels but also to increase the tumor blood perfusion and remodel the TME by reconstructing the aberrant blood vessels within a specific time frame after the medication initiation, so as to relieve hypoxia and improve drug transportation. This is the so-called “vascular normalization” effect and the concept of “window”, which is a key mechanism to exert the synergistic effect of AT combined with RT and chemotherapy.

As one of the representative drugs of AT, RHES exhibits an anti-tumor effect by inhibiting the migratory capacity of endothelial cells involved in neovascularization and has gained extensive clinical application for various malignant tumors, including NPC, demonstrating remarkable efficacy and safety (18–20). Yin et al. (10) analyzed the efficacy and safety of concurrent chemoradiotherapy (CCRT) plus RHES versus CCRT alone in locally advanced nasopharyngeal carcinoma (LANPC), and the survival data of the CCRT + RHES and CCRT groups were as follows: the 3-year progression-free survival (PFS) rates were 81.4% and 63.6% (*p* = 0.034); the 3-year distant metastasis-free survival (DMFS) rates were 88.3% and 77.3% (*p* = 0.049); the corresponding complete remission rates were 100% and 80.0% for lymph node necrosis (*p* = 0.001). These results reveal that CCRT + RHES significantly prolonged 3-year PFS and DMFS in LANPC, and the addition of RHES can enhance the regression of lymph node with necrosis. Moreover, the combined therapy of CCRT + RHES did not increase adverse effect.

In an A549 lung adenocarcinoma xenograft murine model, Li et al. (21) found that RHES induced “vascular normalization” effect in the tumor, which occurred within a specific time window from the 4th to the 10th day post-administration. Peng et al. (22) reported that RHES may normalize tumor vessels in xenografted human NPC models, with the “vascular normalization window” occurring approximately 3 to 7 days after administration. These researchers suggested that the mechanism underlying vascular normalization may be associated with the equilibrium between pro-angiogenic factors and anti-angiogenic factors, which is regulated by RHES.

Currently, histopathological criteria such as microvascular density (MVD), basal membrane thickness, and pericyte coverage rate remain the gold standards for evaluating tumor angiogenesis and tumor vascular normalization (23, 24). However, owing to its invasive nature, over-reliance on accurate sampling, and one-sidedness, evaluating vascular normalization through histopathology poses significant challenges for clinicians. With the advancement of functional imaging techniques, an increasing number of clinicians are inclined to utilize noninvasive and repeatable methods such as computed tomography perfusion imaging (CTPI), CEUS, MRI, and PET to indirectly assess tumor vascular normalization (25). Jiang et al. (26) utilized CTPI combined with ^99^MTCHL91 hypoxic SPECT/CT to assess the vascular normalization effect induced by RHES in non-small cell lung cancer (NSCLC). The results showed that, among the 10 NSCLC patients who received RHES treatment, capillary permeability surface initially decreased and then increased after treatment initiation, reaching its lowest point on the fifth day. Similarly, as a key marker of hypoxic imaging using SPECT/CT, the tumor-to-normal tissue radioactivity ratio revealed a similar trend. In contrast, tumor blood flow initially increased and subsequently decreased following treatment initiation, with the highest value observed on the fifth day. However, in the five patients without RHES treatment, these parameters remained unchanged. Therefore, Jiang et al. concluded that RHES had a normalizing effect on tumor blood vessels in NSCLC patients and that this “window” of vascular normalization appeared immediately upon starting RHES treatment and lasted for approximately 1 week, during which tumor blood perfusion increased and hypoxia improved.

Compared to other imaging methods, CEUS has several advantages: it does not involve radiation, offers good repeatability and real-time capability, and is cost-effective. The most significant advantage of CEUS lies in its utilization of a contrast agent composed of microbubbles with a diameter ranging from 3 to 5 µm. Unlike the iodinated and gadolinium contrast agents used in MRI and CT scans, these microbubbles are similar in size to red blood cells and belong to pure blood pool contrast agents. Consequently, they can be specifically confined within microvessels without diffusing into tissue space. This characteristic allows for the avoidance of interference caused by extravasation of the contrast agent while enabling clear and sensitive monitoring of low-speed microcirculation blood perfusion (27).

According to the introduction provided by the European Federation of Societies for Ultrasound in Medicine and Biology (EFSUMB) (28), PI, MTT, and TTP are all listed as hemodynamic parameters. PI is defined as the maximum value of ultrasonic signal intensity, which is closely associated with the number of microbubbles in the blood vessel and reflects the blood volume of the microvessels within the ROI. TTP is defined as the time taken for the signal to reach its maximum value from its base value. MTT is defined as the average time it takes for all ultrasound contrast agents to pass through ROI. Both TTP and MTT indirectly reflect the blood flow velocity within microvessels in ROI. A comprehensive analysis of these three parameters’ dynamic changes can aid in understanding the alterations in tumor blood perfusion. Liang et al. (29) reported that CEUS parameters indirectly reflect MVD and tumor volume using a subcutaneous transplanted NPC model in nude mice, providing valuable information on angiogenesis and tumor growth. VEGF may play a role in promoting angiogenesis of NPC. Furthermore, Ling et al. (30) conducted a clinical study reporting that imaging perfusion patterns along with quantitative parameters obtained from CEUS, such as PI and TTP, provide high sensitivity and specificity when distinguishing between benign nodules and metastatic nodules located within NPC patients’ necks. These aforementioned studies indicate that CEUS holds significant potential for clinical application in evaluating tumor angiogenesis and blood perfusion.

In a previous study, CEUS was utilized for continuous monitoring of ultrasonic signal variations in the primary tumors located in the nasopharynx of NPC patients before and after AT with RHES. By analyzing the dynamic changes in CEUS parameters, significant alterations were observed within 5 days following initiation of RHES treatment, including an initial increase followed by attenuation in PI, as well as shortening and subsequent prolongation of TTP and MTT in the primary nasopharyngeal tumors. Conversely, no changes were noted in these parameters among the control group. These findings indicated that RHES induces vascular normalization within a specific period following treatment initiation, leading to increased blood perfusion in nasopharyngeal primary tumors. The underlying mechanisms through which RHES induced dynamic changes in CEUS parameters are as follows (31): upon initiation of RHES treatment, VEGF signaling is suppressed, resulting in remodeling of abnormal tumor blood vessels. This leads to thickening of the basement membrane and increased pericyte coverage. Consequently, blood vessel function improves, thereby reducing surface permeability and resistance to fluid and macromolecule flow. As a result, there is an increase in blood perfusion and volume within the neck tCNN along with accelerated blood flow velocity. These morphological and functional vascular changes are reflected accordingly on CEUS as enhanced PI accompanied by reduced TTP and MTT.

Does AT with RHES improve the blood perfusion of the CNN? The answer to this question may be the key to exert the synergistic effect of AT combined with RT and chemotherapy in NPC patients with CNN and improve their outcomes. However, the lack of blood perfusion is the hallmark pathological feature of the metastatic CNN, and the degree of vascular abnormality is necessarily much worse than that of primary tumors in the nasopharynx (5, 6). Therefore, on the basis of previous studies, we conducted this current study.

In this current study, compared to the day before AT with RHES, it was observed that the CEUS parameters (PI, TTP, and MTT) of the tCNN exhibited significant changes on day 5 after treatment initiation. In most patients, PI of the tCNN showed a significant increase, while TTP and MTT demonstrated significant decreases. These results indicate an augmentation in blood volume and blood flow velocity within the microvessels of CNN following AT, suggesting that RHES may normalize the abnormal vessels and improve blood perfusion in CNN of NPC patients, thereby alleviating hypoxia and increasing drug delivery efficiency. Furthermore, according to previous reports, the common adverse effects of RHES include allergy, headache, arrhythmia, nausea, and fever. However, no adverse effect occurred in this study, which may be related to the limited sample size and short observation time.

As a preliminary study, it has limitations such as a small sample size, the lack of external control, a short observation time, and the lack of long-term efficacy data. Because of these limitations, the results of this research must be interpreted with caution. Further prospective randomized controlled trials with a large sample size and long-term follow-up data are warranted to verify our findings.

## Conclusions

In conclusion, the results of this study suggest that AT with RHES may improve blood perfusion in the CNN of NPC, thus providing valuable insights for the clinical application of AT combined with RT and/or chemotherapy in NPC patients with CNN. Moreover, CEUS as a noninvasive and real-time monitoring method may be suitable for clinically evaluating tumor blood perfusion changes.

## Data Availability

The original contributions presented in the study are included in the article/supplementary material. Further inquiries can be directed to the corresponding author.
